# Antidiabetic and antioxidant effects of catalpol extracted from *Rehmannia glutinosa* (Di Huang) on rat diabetes induced by streptozotocin and high-fat, high-sugar feed

**DOI:** 10.1186/s13020-016-0096-7

**Published:** 2016-05-11

**Authors:** Huifeng Zhu, Yuan Wang, Zhiqiang Liu, Jinghuan Wang, Dong Wan, Shan Feng, Xian Yang, Tao Wang

**Affiliations:** College of Pharmaceutical Sciences and Traditional Chinese Medicine, Southwest University, Chongqing, 400715 China; Chongqing Engineering Research Center for Pharmacological Evaluation, POB 400715, Chongqing, China; Department of Pharmacy, The First People’s Hospital of Neijiang, Neijiang, 641000 China; Department of Emergency, The First Affiliated Hospital of Chongqing Medical University, POB 400016, Chongqing, China

## Abstract

**Background:**

Diabetes, associated with hyperlipidemia and oxidative stress, would lead to an increased production of reactive oxygen species. *Rehmannia glutinosa* (Di Huang) is widely used to nourish *yin*, invigorate the *kidney* (*shen*), and treat *xiao ke* (a diabetes-like syndrome in Chinese medicine). This study aims to investigate the antidiabetic and antioxidant effects of catalpol from *R. glutinosa* on rat diabetes induced by streptozotocin (STZ) and high-fat, high-sugar feed.

**Methods:**

Rats (eight rats in each group at least) were induced diabetes by an initial high-fat high-sugar feed for 3 weeks, followed by an intraperitoneal injection of STZ (30 mg/kg) for 3 days, and rats were fasted overnight before treatments. Catalpol at a dose of 0, 5, 10, 20 or 50 mg/kg was administrated through bolus intravenous injection to the experimental rats to find the most effective anti-hyperglycemic dose of catalpol to further study body weight loss, water intake, and food intake. The most effective catalpol dose was given to the diabetic model rats with hyperlipidemia, and the levels of blood sugar, plasma total cholesterol (TC), triglyceride (TG), and high-density lipoprotein cholesterol (HDL-C) were measured after catalpol administration once a day for 2 weeks. An oral glucose challenge test (OGCT) was performed after above experiments in which the most effective dose of catalpol has been determined. Levels of glutathione peroxidase (GSH-PX), catalase (CAT), superoxide dismutase (SOD), and malondialdehyde (MDA) were measured by corresponding reagent kits and morphological changes of the pancreas were observed with histopathological examination using H&E stain.

**Results:**

Catalpol at a dose of 50 mg/kg ameliorated body weight loss and increased water and food intake. Catalpol also attenuated the increase of plasma TC (*P* = 0.0067) and TG (*P* = 0.0084) and increased HDL-C (*P* = 0.0336). The OGCT revealed that catalpol reduced the increase of plasma glucose. The activities of antioxidative enzymes (SOD, *P* = 0.0037; GSH-PX, *P* = 0.0066; CAT, *P* = 0.005) were enhanced and MDA was reduced (*P* = 0.003). Furthermore, catalpol reduced the morphological impairment of the pancreas.

**Conclusion:**

Catalpol protected against STZ-induced diabetes with high-fat and high-sugar feed with ameliorated structural impairment of the pancreas and restored balance between oxidative enzymes and antioxidative enzymes.

## Background

Diabetes mellitus is a heterogeneous metabolic disorder characterized by chronic hyperglycemia. It is associated with long-term complications and results in various bodily dysfunctions [1]. Diabetes often leads to the vascular complications of coronary artery disease and cerebrovascular disease and can cause renal failure, blindness, limb amputation, neurological complications, and premature death. With rapid therapeutic advancements, novel treatments with fewer side effects have become more feasible for long-term management of this disorder [2].

*Rehmannia glutinosa* (Di Huang) is a widely used herb in Chinese medicine (CM) that belongs to the Scrophulariaceae family. It has a high medicinal value and is taken to nourish *yin* and invigorate the *kidney* in CM [3]. *R. glutinosa* is often used to treat *xiao ke* [4], both *xiao ke* and diabetes patients experience the “3Ps”: polyuria (frequent urination), polydipsia (increased thirst), and polyphagia (increased hunger). CM theory attributes *yin* deficiency to diabetes [4] and *R. glutinosa* is often clinically used to ameliorate patients’ symptoms.

Catalpol, an iridoid glucoside of *R. glutinosa*, has many biological effects, including anticytotoxic and anti-inflammatory properties. Catalpol protected mesencephalic neurons from 1-methyl-4-phenylpyridinium [MPP(+)]-induced oxidative stress [5]. It also protected against hydrogen peroxide-induced apoptosis by preventing cytochrome c release and inactivating the caspase cascade, [6] against hydrogen peroxide-induced oxidative damage [7], and against ischemia-induced damage in astrocytes. Moreover, in an animal model, catalpol treatment ameliorated cognitive deficits and attenuated oxidative damage in brains of aging mice induced by d-galactose, increased the activities of SOD and GSH-PX, and reduced MDA levels in the liver and spleen [8].

Recent works have demonstrated catalpol’s anti-hyperglycemic effect in streptozotocin (STZ)-induced [2] and alloxan-induced rat diabetes [9, 10]. Catalpol could increase glucose use to lower plasma glucose in diabetic rats lacking insulin [2] through increasing beta-endorphin secretion from the adrenal gland in STZ-diabetic rats [11]. Moreover, oral supplementation of catalpol ameliorates diabetic encephalopathy by attenuating oxidative stress and increasing nerve growth factor concentration [1]. It ameliorated diabetic nephropathy [12] in rats by reducing the expression of pro-inflammatory mediators, such as monocyte chemotactic protein-1, tumor necrosis factor-alpha, inducible nitric oxide synthase, and receptors for advanced glycation endproducts (AGE). Catalpol suppressed AGE-induced phosphorylation of mitogen activated protein kinases, degradation of IkappaBalpha, and the nuclear localization of nuclear factor-kappaB [13]. Most studies of the effects of catalpol on diabetes mellitus have used normal diets in experimental models of chemical diabetes. However, type 2 diabetes mellitus is usually accompanied by glycolipid metabolism disorders and the effect of catalpol on type 2 diabetes mellitus with hyperlipid syndrome after a high-fat diet is still unknown.

This study aims to investigate the antidiabetic and antioxidant effects of catalpol from *R. glutinosa* on rat diabetes induced by STZ and high-fat, high-sugar feed. STZ and alloxan have been used to induce a diabetes mellitus model and damage the pancreas. Oxidative stress produced by STZ and alloxan is involved in diabetes mellitus and catalpol seems to have good anti-oxidative stress properties. In the present study, STZ-induced diabetes mellitus with a high-sugar and high-fat diet was used to imitate human type 2 diabetes mellitus. We investigated the effects of catalpol on glucose and fat metabolism in STZ-induced diabetic rats and measured oxidative enzyme and antioxidative enzyme plasma levels to explore the possible mechanisms of catalpol on type 2 diabetes mellitus.

## Methods

### Materials

STZ was obtained from Sigma-Aldrich Inc. (Sigma, S0130, USA). Analytical grade catalpol (product >99 % purity) was purchased from Liubobainiao Biotechnology Co., Ltd (Shijiazhuang, China). Blood glucose Span Diagnostic kit and Jinque test strips was obtained from Shanghai MicroSence Inc. (Shanghai, China). Blood lipid commercially available kits, CAT, GSH-PX, SOD and MDA commercially available kits were from Nanjing Jiancheng Bioengineering Institute (China).

### Experimental animals and feeds

One hundred twenty male Wistar rats (200–250 g) were procured from the Experimental Animal Center, Chongqing Medical University, China. The rats were arbitrarily divided into four groups: normal group (n = 8), high-sugar and high-fat feed (high-fat, n = 8) group, STZ-diabetic animals with high-sugar and high-fat feed (STZ-fat, n = 12), and STZ-diabetic animals with high-sugar and high-fat feed treated with catalpol (STZ-fat-cat, n = 12).

Rats were kept in an air-conditioned room maintained at a constant temperature (24–26 °C) and humidity (50–60 %) under a 12-h light/dark cycle (07:00 on and 19:00 off). Standard rat feed and water were provided ad libitum. The rats were allowed to acclimatize to the laboratory environment for 7 days before the start of the experiment. All experimental procedures were conducted in conformity with institutional guidelines for the care and use of laboratory animals in China (Permit: SCXK 2002A040), and the international guidelines on the ethical use of animals (NIH publications No. 80–23, revised 1996).

The high-sugar, high-fat diet was comprised of normal diet 54 kg, sucrose 16.5 kg, lard 8.31 kg, egg yolk 4.14 kg, and salt 83.4 g, with a total combined weight of 83.784 kg [14, 15]. The normal diet was purchased from Beijing China Fukang Biotechnology Co., Ltd (Beijing, China). The main components of the normal diet were crude protein ≥18 %, crude fat ≥4 %, crude fiber ≥5 %, crude ash ≤8 %, moisture ≤10 %, lysine ≥0.82 %, calcium 1–1.8 %, phosphorus 0.6–1.2 %, and salt 0.2–0.8 %.

### Induction of diabetes in rats

Diabetes was induced by an initial high-sugar and high-fat feed for 3 weeks, followed by an intraperitoneal injection of STZ (30 mg/kg) for 3 days. STZ was dissolved in a freshly prepared 0.01 M citrate buffer (pH 4.5). The normal control group was injected with buffer alone. Rats with blood glucose ≥16.7 mmol/L for 3 weeks were considered as diabetic [16] and the death rates for modeling of STZ-fat were 22–28 %.

### Experimental design and catalpol treatment of rats

At the first stage, catalpol was injected into the rat tail vein at a dose of 0, 5, 10, 20, or 50 mg/kg. Blood was collected after catalpol treatment for 14 days to assess the blood glucose-lowering effect of catalpol.

At the second stage, the most effective dose of catalpol was selected for further study. The plasma lipid level was detected in the normal, high-sugar and high fat feed, STZ-fat, and STZ-fat-cat groups at 14 days after treatment with catalpol. The plasma in each group was collected and then antioxidative enzymes such as GSH-PX, SOD, CAT, and the MDA oxidative products were detected with corresponding kits. Oral glucose challenge tests (OGCT) were then performed on experimental rats using a Roche blood glucose monitor (Roche Diagnostics, Rotkreuz, Switzerland.) with Jinque test strips. During the experiment, body weight, food intake, and water intake were recorded every 2 days. Finally, a splenic portion of pancreatic tissue was removed immediately after sacrifice to observe morphological changes of the pancreas.

### Determination of plasma glucose

Blood samples were collected from the inner canthus using a capillary tube under chloral hydrate anesthesia at each time point. Concentration of blood glucose was determined by a glucose meter (Roche Diagnostics, Rotkreuz, Switzerland) with Jinque test strips (Span Biotech Ltd, India).

### Plasma collection and biochemical determination

Blood samples were collected from the inner canthus using a capillary tube under chloral hydrate anesthesia after catalpol treatment for 14 days. The samples were centrifuged at 2810×*g* for 10 min at 4 °C within 1 h after collection, and then supernatants were collected. The concentration of plasma total cholesterol (TC), triglyceride (TG), and high-density lipoprotein cholesterol (HDL-C); the activities of SOD, GSH-PX, CAT and the concentration of MDA were determined by a series of commercially available kits including SOD, GSH-PX, CAT, and MDA kits (Nanjing Jiancheng Bioengineering Institute, China) according to the manufacturers’ instructions.

### Oral glucose challenge test

An OGCT was performed according to a method previously described with minor modifications [17–19]. Briefly, catalpol at the most effective dose, or the same volume of vehicle, was injected intravenously into rats for 14 days. Then a glucose dose of 2.5 g/kg was intragastrically administrated to the rats. Blood samples were collected sequentially from the tail vein before (0 h) and 0.5, 1, 2, 3, 5, 7, and 9 h post challenge. Plasma glucose changes at the indicated time were determined with a glucose meter (Accu-Chek, Switzerland) and Jinque test strips.

### Histological evaluation of pancreas with hematoxylin and eosin (H&E) staining

A splenic portion of pancreatic tissue was removed immediately after sacrifice and rinsed with ice-cold saline. The tissue samples were fixed in 4 % buffered neutral paraformaldehyde solution overnight, embedded in paraffin, and deparaffinized using standard procedures [20, 21]. Thin Sections (5 µm) were dewaxed, dehydrated in a graded series of ethanol, and rehydrated, then stained with H&E for light microscopic examination (Leica, Germany). All histological analyses were performed in arbitrarily selected fields in sections by two investigators blinded to the identity of the treatment groups.

### Statistical analysis

All data were analyzed by the SPSS statistical software (version 13.0, SPSS, Chicago, IL, USA), with *P* < 0.05 values were considered as statistically significant. Results were expressed as mean ± standard deviation (SD). Comparisons between the groups were assessed by paired-samples *t* test, and *P* value correction for multiple group comparison by LSD-*t* test. Dose-dependent manner of catalpol’s hyperglycemic action was visually determined from a dose–response curve.

## Results

### Dose-dependent anti-hyperglycemic action of catalpol

**Fig. 1:**
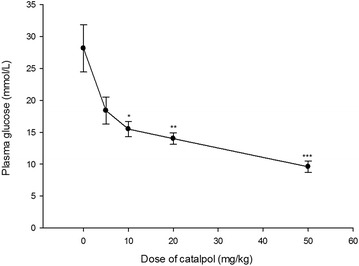
Anti-hyperglycemic action of catalpol for 2 weeks in STZ-diabetic rats with high-sugar and high-fat diet. Values (mean ± SD) were obtained from eight animals through intravenous injection (iv) of catalpol at the indicated dose. *P = 0.02, **P = 0.005 and ***P < 0.001 as compared with no treatment values (0 mg/kg)

Catalpol at higher doses lowered plasma glucose concentrations more effectively in STZ-fat-diabetic rats. A dose-dependent increase of anti-hyperglycemic activity was observed in STZ-fat-diabetic rats upon intravenous injection of catalpol at the dose range 10–50 mg/kg for 2 weeks (Fig. 1). There was no additional effect of catalpol with an increase in dosage beyond 50 mg/kg. The minimal and maximal plasma glucose-lowering activities of catalpol in STZ-fat-diabetic rats were 21.7 ± 1.3 % at 10 mg/kg and 65.8 ± 3.07 % at 50 mg/kg.

### Effects of catalpol on body weight

**Fig. 2:**
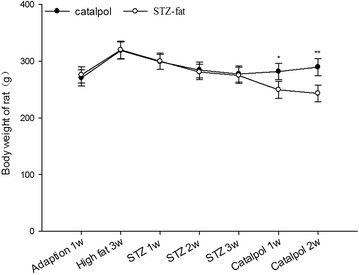
Effects of catalpol on body weight gains of rats. After given high-sugar high-fat food for 3 weeks, and STZ induced STZ-fat-diabetic rats successfully for 3 weeks, a solution of catalpol at 50 mg/kg or the same volume of vehicle was injected intravenously into rats. Changes of body weight at the indicated time point were compared between the catalpol-treated group (*solid circles*) and the vehicle-treated group (*open circles*). Values (mean ± SD) of each group were obtained from eight animals. *P = 0.047 and **P = 0.009 as compared with vehicle-treated group at the same time

As shown in Fig. 2, after adaptation feed for 1 week, and then high-fat and high-sugar feed for 3 weeks, the initial body weight and the body weight increase in each group were similar. After administration of STZ, the body weight of the STZ-fat-diabetic rats continued to reduce. At the end of the study, the diabetic rats in all groups had lost body weight even when compared with their weight after high-fat and high-sugar feeding for 3 weeks (*P* = 0.047). However, the final body weight in the catalpol group was significantly greater than the body weight in the STZ-fat-diabetic rats (*P* = 0.009) and was close to the starting weight.

### Effects of catalpol on feed consumption

**Fig. 3:**
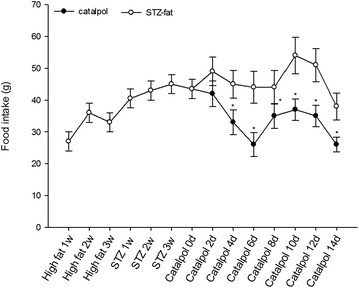
Effect of catalpol on food consumption of rats. After given high-fat high-sugar food for 3 weeks, and STZ induced STZ-fat-diabetic rats successfully for 3 weeks, a solution of catalpol at 50 mg/kg or the same volume of vehicle was injected intravenously into rats. Changes of food intake at the indicated time point were compared between the catalpol-treated group (*solid circles*) and the vehicle-treated group (*open circles*). Values (mean ± SD) of each group were obtained from eight animals. *P < 0.05 as compared with vehicle-treated group at the same time

After adaptation feed for 1 week, and high-fat and high-sugar feed for 3 weeks, food intake starting in the high-fat and high-sugar feed period increased quickly in the first 2 weeks, then began to decrease, and increased obviously when STZ was given to induce diabetes in the STZ-fat-diabetic group (Fig. 3). When catalpol was given for 4 days, the food intake obviously reduced compared with that of STZ-fat-diabetic rats administered with same volume of vehicle (*P* = 0.045), and continued to reduce for 14 days after catalpol treatment (*P* = 0.020–0.045).

### Effects of catalpol on water intake

**Fig. 4:**
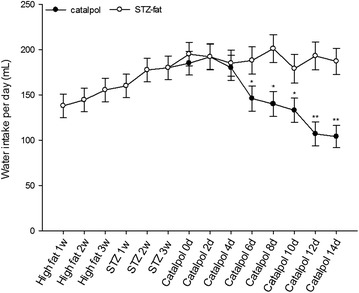
Effect of catalpol on water intake of rats. After given high-fat high-sugar food for 3 weeks, and treatment by STZ for 3 days induced STZ-fat-diabetic rats successfully for 3 weeks. A solution of catalpol at 50 mg/kg or the same volume of vehicle was injected intravenously into rats. Changes of water intake at the indicated time point were compared between the catalpol-treated group (*solid circles*) and the vehicle-treated group (*open circles*). Values (mean ± SD) of each group were obtained from eight animals. *P < 0.05 and **P < 0.01 as compared with vehicle-treated group at the same time

As shown in Fig. 4, after adaptation feed for 1 week, and then high-fat and high-sugar feed for 3 weeks, water intake increased quickly starting in the high-fat and high-sugar feed period, and continued to increase for 3 weeks after STZ-fat-diabetes was successfully induced. After catalpol treatment for 6, 8, and 10 days, the water intake obviously reduced compared with water intake in the STZ-fat-diabetic rats administered with the same volume of vehicle (*P* = 0.05, 0.034, 0.048, respectively). At 12 and 14 days, water intake reduced significantly compared with the control rats (*P* = 0.0087, 0.0089, respectively).

### Anti-hyperglycemic action of catalpol in rat diabetes induced by STZ with high-sugar and high-fat feed

**Fig. 5:**
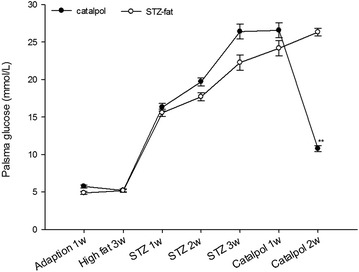
Anti-hyperglycemic action of catalpol for 2 weeks in STZ-diabetic rats with high-sugar and high-fat feed. Values (mean ± SD) were obtained from eight animals through intravenous injection (iv) of catalpol at 50 mg/kg dose. *P = 0.0031 as compared with non-treated group (*saline*)

No group differences in plasma sugar were found before STZ administration (Fig. 5). Plasma sugar reached 16.7 mmol/L for 1 week after STZ was given, and plasma sugar in each group became gradually elevated. However, plasma sugar decreased significantly after catalpol (50 mg/kg) treatment for 2 weeks, compared with that of the STZ-fat-diabetic rats (*P* = 0.0031), and plasma sugar recovered to the level before STZ administration.

### Anti-hyperlipid action of catalpol

**Fig. 6:**
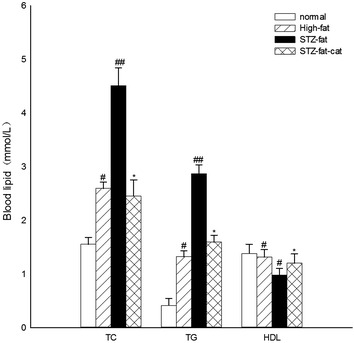
Anti-hyperlipid action of catalpol for 2 weeks in STZ-diabetic rats with high-sugar and high-fat feed. Values (mean ± SD) were obtained from eight animals through intravenous injection (iv) of catalpol at 50 mg/kg dose. ^#^ *P* < 0.05 vs. normal; ^##^ *P* < 0.01 vs. high fat group and *P < 0.05 vs. STZ-fat group

As shown in Fig. 6, TC and TG were obviously elevated in rats given the high-fat and high-sugar diet for 3 weeks compared with the normal group (*P* = 0.036, 0.04, respectively) and HDL-C decreased significantly (*P* = 0.045). After STZ administration, TC and TG increased remarkably (*P* = 0.0067, 0.0058, respectively) compared with those of the high-fat group and the HDL-C in the high-fat group was reduced compared with that of the normal group (*P* = 0.044). STZ-fat-diabetic rats treated with catalpol (50 mg/kg) showed significantly attenuated TC (*P* = 0.0067) and TG (*P* = 0.0084) compared with the high-fat group. The level of HDL-C obviously increased (*P* = 0.0336), although TC, TG, and HDL-C levels did not all return to normal.

### Effects of catalpol on the oral glucose challenge test in STZ-fat-diabetic rats

**Fig. 7:**
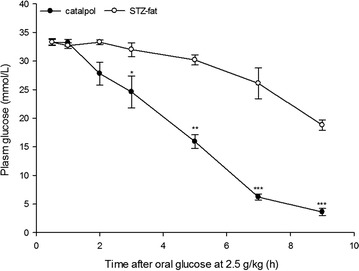
Effect of catalpol on plasma glucose levels in STZ-fat diabetic rats in an oral glucose challenge test. A solution of catalpol at 50 mg/kg or the same volume of vehicle was intragastrically administrated into rats. Then, a glucose dose of 2.5 g/kg was intragastric administration into each rat. Changes of plasma glucose at the indicated time were compared between the catalpol-treated group (*solid circles*) and the vehicle-treated group (*open circles*). Values (mean ± SD) of each group were obtained from eight animals. *P < 0.05, **P < 0.01, and ***P < 0.001 as compared with vehicle-treated group at the same time

An OGCT was then carried out to determine the glucose clearance. The plasma glucose concentration was elevated in both vehicle- and catalpol-treated rats 30 min after oral glucose administration (Fig. 7). However, there were no statistically significant differences in plasma glucose concentration between the catalpol group and the vehicle group within 3 h after treatment. The increased plasma glucose induced by the oral glucose was significantly lower in the rats at 3 h in the catalpol group compared with the vehicle-treated rats (*P* = 0.046), and this pattern continued for 5 h (*P* = 0.0076), 7 h (*P* < 0.001), and 9 h (*P* < 0.001), finally returning to normal. It means that significantly higher plasma glucose concentrations were detected in the vehicle-treated group at 3, 5, 7, and 9 h time points as compared to that in the catalpol group. This suggested that catalpol could enhance glucose use in vivo.

### Effects of catalpol on oxidative stress in STZ-treated diabetic rats

**Fig. 8:**
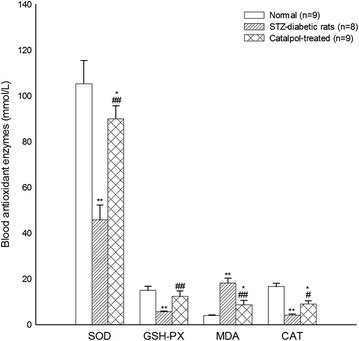
Effect of catalpol on the activities of antioxidant enzymes and the concentration of MDA in the plasma. *P < 0.05, **P < 0.01 vs. normal group, ^#^ *P* < 0.05, ^##^ *P* < 0.01 vs. STZ-treated group

The activities of antioxidative enzymes and the MDA plasma concentrations were shown in Fig. 8. There were no significant differences in oxidative stress between the high-fat and the normal group, although a rising trend in MDA and a decreasing trend in GSH-PX, SOD, and CAT activity were found in the high-fat group. The activities of GSH-PX, SOD, and CAT (*P* = 0.008, 0.002, 0.007, respectively) in STZ-treated diabetic rats were significantly lower than in the normal control rats and high-fat rats, while the concentration of MDA (*P* = 0.006)in STZ-treated diabetic rats was significantly greater than in the normal control rats. Catalpol significantly improved the activities of antioxidative enzymes and decreased MDA concentration.

### Effect of catalpol on morphological changes of the pancreas

**Fig. 9:**
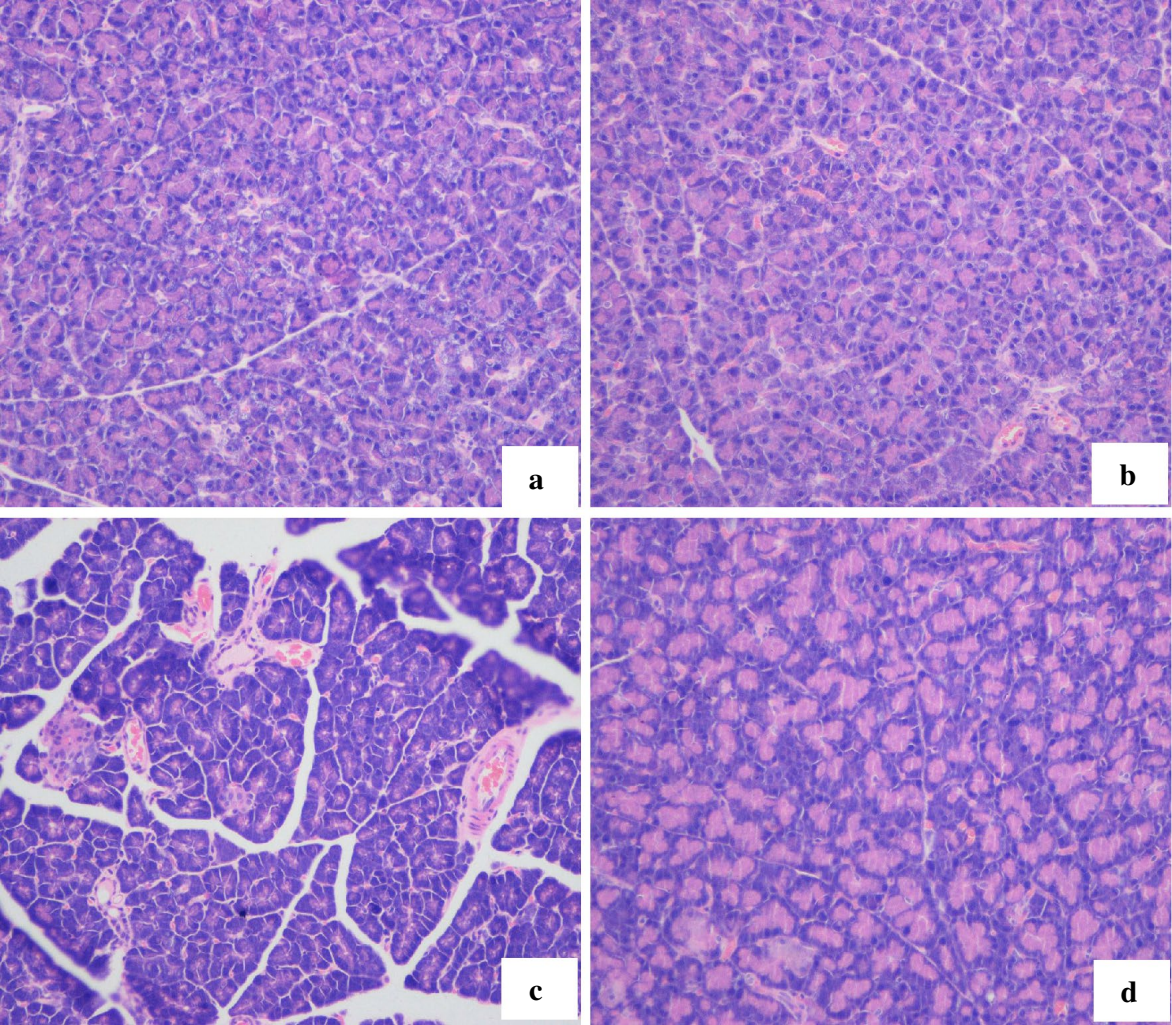
Representative light microscopic appearances of the pancreas stained with H&E (200x) (**a**–**d**). **a** Normal histological changes were observed in the normal group on day 14. **b** Only minimal histological changes were observed in the control (high-fat and high-sugar) group on day 14. **c** The pancreatic ductal hypertension STZ-fat-diabetic rats group, infiltration of inflammatory cells were found in the interlobular and periductal areas on day 14. The pancreas was atrophic, and fibrosis and inflammatory cells were markedly distributed in the interlobular and interlobular areas. **d** In contrast, distributions of inflammatory cells and fibrosis were slightly noted Leukocyte infiltration in the catalpol group on day 14. The *arrows* represent inflammatory cells infiltration

Only minimal pancreatic histological changes were observed in the high-fat group, compared with normal pancreatic tissue (Fig. 9a). Minimal inflammatory cells were infiltrated into the intralobular area and around the pancreatic ducts (Fig. 9b). However, in the STZ-fat-diabetic group, the pancreatic ducts were hypertense and markedly dilated, there was much infiltration of inflammatory cells in the intralobular and periductal areas, the exocrine glands of the pancreas were atrophic, and the fibrotic areas were markedly distributed around the pancreatic ducts in the interlobular and intralobular areas (Fig. 9c). In contrast, distribution of fibrosis and inflammatory cells was markedly attenuated in the STZ-fat-cat group and there was no pancreatic ductal hypertension and dilation (Fig. 9d).

## Discussion

In this study, STZ-induced type 2 diabetes with a high-fat and high sugar diet was used to study the antidiabetic and antioxidant effects of catalpol. STZ is synthesized by *Streptomycetes achromogenes* and is used to induce both insulin-dependent and non-insulin-dependent diabetes mellitus based on the STZ dose [22, 23]. The most frequently used single intravenous dose to induce insulin-dependent diabetes mellitus in adult rats is between 40 and 60 mg/kg by body weight [24]. STZ is also efficacious after intraperitoneal administration of a similar or higher dose, but a single dose below 40 mg/kg body weight may be ineffective [25]. STZ can also be given in multiple low doses. In this paper, rat diabetes was induced by a small dose of STZ (30 mg/kg) for 3 days after the rats were given high-fat and high-sugar feed for 3 weeks; this modeled more closely human type 2 diabetes because it accompanied the lipid abnormality. Before STZ administration, rats were given a high-sugar and high-fat diet for 3 weeks to induce fat and lipid abnormities, and their fasting blood sugar was near normal (less than 7 mmol/L). The plasma sugar and lipids were high after STZ administration for 2 weeks. STZ-diabetic body weight obviously decreased and was accompanied by increased thirst, frequent urination, and increased hunger. These changes indicated diabetes was successfully induced.

We found that catalpol had positive effects on type 2 diabetes. A dose-dependent anti-hyperglycemic action was observed for intravenous injection of catalpol at the dose range 10–50 mg/kg for 2 weeks. An increase in dosage beyond 50 mg/kg produced no additional effects. We chose a catalpol dose of 50 mg/kg for further study. The data showed that diabetic rats consumed more food and water and lost body weight. Interestingly, 50 mg/kg catalpol treatment for 2 weeks significantly ameliorated body weight loss and attenuated water intake and food consumption. Although we did collect urine or measure urine volume in each group, STZ-fat-diabetes rats became increasingly thinner, and the padding was often wet, which meant diuresis was markedly obvious in STZ-fat-diabetic rats; however, catalpol obviously ameliorated these changes.

As a glucose load test, the OGCT was used to test glucose clearance, which reflects pancreatic β cell function. Catalpol treatment 3 h after oral glucose administration obviously reduced plasma glucose and levels returned to normal in 6 h. The OGCT results showed that catalpol treatment improved diabetic rats’ ability to adjust blood sugar, perhaps through improved islet β cell function. Histological evaluation of the pancreas showed clearly (Fig. 9c) that the pancreatic tissue was atrophic with the presence of fibrosis. There was infiltration of inflammatory cells and these were markedly distributed in the interlobular and intralobular areas, but there was minimal distribution of inflammatory cells and fibrosis in the catalpol group. These results indicate that catalpol might facilitate glucose uptake, which is in accordance with previous findings [2].

Diabetes can cause glucose-lipid metabolism disturbance. Many diabetes patients show long-term abnormal lipid metabolism. In this study, the animal model induced by STZ with high-sugar and high-fat feed was close to human diabetes type 2. The rat model indicated that high-sugar and high-fat food with STZ produced type 2 diabetes accompanied by lipid metabolism disturbances with increases in TC and TG and decreases in HDL-C. Catalpol lowered plasma TC and TG and increased HDL-C (Fig. 5) when the blood sugar was lowered (Fig. 6). Catalpol not only ameliorated abnormal glucose tolerance, but also relieved the glucose-lipid metabolism disturbance.

Oxidative stress plays an important role in diabetes mellitus induced by STZ. STZ enters β cells via a glucose transporter and causes alkylation of DNA. DNA damage induces the activation of poly adenosine diphosphate-ribosylation, a process that is more important for the diabetogenicity of STZ than DNA damage itself [26]. Poly adenosine diphosphate-ribosylation leads to the depletion of cellular nicotinamide adenine dinucleotide and adenosine triphosphate. Enhanced adenosine triphosphate dephosphorylation after STZ treatment supplies a substrate for xanthenes oxidase resulting in the formation of superoxide radicals, hydrogen peroxide and hydroxyl radicals. Furthermore, STZ liberates toxic amounts of nitric oxide that inhibit aconitase activity and contribute to DNA damage. STZ can cause β cell necrosis [22, 23, 26]. In addition to a plasma lipid and sugar increase in STZ-fat-diabetic rats, the present findings show that endogenous antioxidants, such as SOD, GSH-PX, and CAT, were decreased and MDA levels were increased, which indicates that oxidative stress was strengthened in STZ-fat-diabetic rats. However, catalpol at 50 mg/kg for 2 weeks significantly reversed the lowered antioxidant levels and the increase of oxidative stress induced by STZ with sugar-lipid abnormities. SOD, GSH-PX, and CAT in plasma increased and MDA levels reduced to recover the balance of endogenous oxidants and antioxidants. The main active ingredients of catalpol, which is an iridoid glucoside, are isolated from the roots of *R. glutinosa* and used for treatment of diabetes and its complications. Furthermore, catalpol is a strong antioxidant [5, 27, 28] used to treat aging, stroke, and demyelination disease. The main effects of catalpol are on endogenous oxidant damage [1, 6, 7, 28] and inflammatory reactions [29, 30]. The antioxidant effects of catalpol found here are in accord with previous findings [1, 6, 7, 28]. However, catalpol possessed a stronger anti-inflammatory action and reduced inflammatory cytokines in senescent mice [29, 30], inhibited lipopolysaccharide plus interferon-gamma-induced inflammatory responses in astrocyte primary cultures [29, 30], and suppressed advanced glycation end-products-induced inflammatory responses through the inhibition of reactive oxygen species [13]. Based on the anti-hyperglycemic effect of catalpol and anti-hyperlipid abnormal coupling with markedly reduced oxidative stress in plasma, morphological pancreatic changes were examined by a light microscope (Fig. 9) after treatment with catalpol. Pancreatic damage induced by STZ was manifested as inflammation: infiltration of inflammatory cells was noted in the hypertension and markedly dilated intralobular and periductal areas. The atrophic, fibrotic, and inflammatory cells were markedly distributed in the interlobular and intralobular areas (Fig. 9c). After treatment with catalpol for 14 days, injury was ameliorated and blood glucose was decreased, and lipid levels also decreased as catalpol attenuated oxidative stress in STZ-fat-diabetic rats. In addition, inflammatory cells and fibrosis were minimal (Fig. 9d) and the pancreatic morphological changes were similar to those of the high-fat, high-sugar group (Fig. 9b). This suggests that catalpol might improve insulin levels in plasma through mitigation of pancreas inflammation induced by STZ and preserve pancreas islet structure and function.

We observed protective effects of catalpol on STZ-fat-diabetic rats. To date, only two studies have investigated the effect of catalpol on STZ-induced diabetes; these have focused on catalpol’s actions on preventing diabetic encephalopathy [1] and lowering plasma glucose through increasing muscle glucose use [2]. To our knowledge, this is the first study to investigate the effect of catalpol on diabetes induced by STZ with a high-sugar and high-fat diet. Catalpol reduced the damage to the pancreas in STZ-fat-diabetic rats. Catalpol’s protective effects are mainly related to decreased oxidative stress and the attenuation of the “3P” symptoms in a severely diabetic animal model. Further mechanisms of action, such as those involving insulin or glycated hemoglobin levels, should be investigated further.

## Conclusion

Catalpol protected against STZ-induced diabetes with high-fat and high-sugar feed with ameliorated structural impairment of the pancreas and restored balance between oxidative enzymes and antioxidative enzymes.

## Abbreviations

STZ: streptozotocin
ALX: alloxan
CM: Chinese medicine
3P: polyuria (frequent urination), increasingly thirsty (polydipsia) and hungry (polyphagia)
TC: plasma total cholesterol
TG: triglyceride
HDL-C: high-density lipoproteincholesterol
H&E: hematoxylin and eosin
OGCT: oral glucose challenge test
GSH-PX: glutathione peroxidase
MDA: malondialdehyde
SOD: superoxide dismutase
CAT: catalase
LPS: lipopolysaccharides
